# Disorder strongly enhances Auger recombination in conductive quantum-dot solids

**DOI:** 10.1038/ncomms3329

**Published:** 2013-09-13

**Authors:** Yunan Gao, C. S. Suchand Sandeep, Juleon M. Schins, Arjan J. Houtepen, Laurens D. A. Siebbeles

**Affiliations:** 1 Optoelectronic Materials Section, Department of Chemical Engineering, Delft University of Technology, Julianalaan 136, 2628 BL Delft, The Netherlands; 2 The Kavli Institute of Nanoscience, Delft University of Technology, Lorentzweg 1, 2628 CJ Delft, The Netherlands

## Abstract

Auger recombination (AR) can be an important loss mechanism for optoelectronic devices, but it is typically not very efficient at low excitation densities. Here we show that in conductive quantum-dot solids, AR is the dominant charge carrier decay path even at excitation densities as low as 10^−3^ per quantum dot, and that AR becomes faster as the charge carrier mobility increases. Monte Carlo simulations reveal that this efficient AR results from charge carrier congregation in ‘Auger hot spots’: lower-energy sites that are present because of energy disorder. Disorder-enhanced AR is a general effect that is expected to be active in all disordered materials. The observed efficient AR is an issue of concern for devices that work at charge carrier densities in excess of ~10^−3^ charge carriers per quantum dot. At the same time, efficient carrier congregation could be exploited for fast optical switching or to achieve optical gain in the near infrared.

## Abstract (web summary)

Understanding Auger recombination is important for building better optoelectronics, as it is a crucial loss mechanism. Gao *et al.* study it in conductive quantum dot solids, and find that it is the dominant charge carrier decay path, even for very low excitation densities.

## Graphical abstract

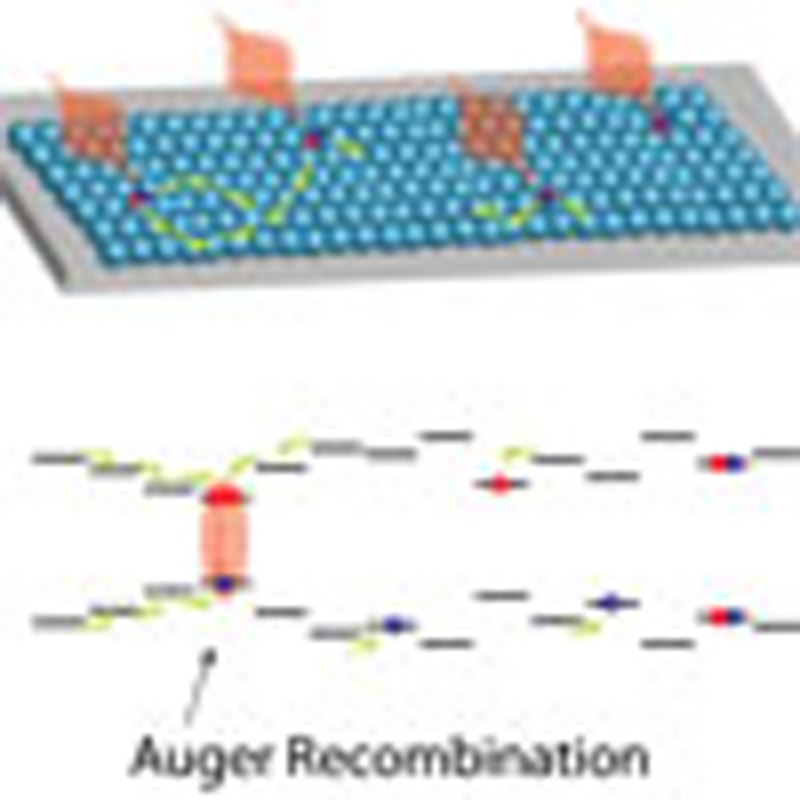

Auger recombination (AR) is a process in which an electron recombines with a hole while transferring the electron–hole pair energy to a third charge carrier. It is well known to occur in (inorganic) semiconductor materials^1^,^2^,^3^. In organic materials AR also occurs, although it is typically referred to as exciton-exciton annihilation^4^,^5^,^6^. AR can be an important loss mechanism for many optoelectronic devices, but as it is a higher-order process it is typically not efficient at low excitation densities. In semiconductor quantum dots (QDs), AR has been long studied and the AR rate has been shown to be very high, leading to typical recombination times for biexcitons of 50 ps^7^. However, as AR requires two excitons, in colloidal dispersions of QDs the process is only observed when more than one photon is absorbed per QD. A natural assumption, and one that is often encountered in the literature, is that AR is also insignificant in films of QDs (QD solids) if the excitation density is <1 per QD^8^,^9^,^10^,^11^.

Here we present photoconductivity and transient absorption (TA) studies on QD solids over a wide range of excitation densities and observe higher-order decay at excitation densities as low as 10^−3^ per QD. This decay is attributed to diffusion-mediated AR. We compare QD films with ligands of different lengths, and concomitantly differing charge carrier mobilities, and demonstrate that AR becomes faster as the mobility increases. From Monte Carlo simulations, we find that this efficient AR stems from charge carrier congregation in low-energy sites that are present because of energy disorder, which we term ‘Auger hot spots’. Disorder-enhanced AR is a general effect that is expected to be active in all disordered materials. The observed efficient AR is an issue of concern for devices that work at charge carrier densities in excess of ~10^−3^ charge carriers per QD, such as QD light-emitting diodes (LEDs) or solar cells under concentrated sunlight. Fast carrier congregation in low-energy sites could possibly be exploited for fast optical switching or to achieve optical gain in the near infrared (NIR).

## Results

### Photoconductance

**Figure 1:**
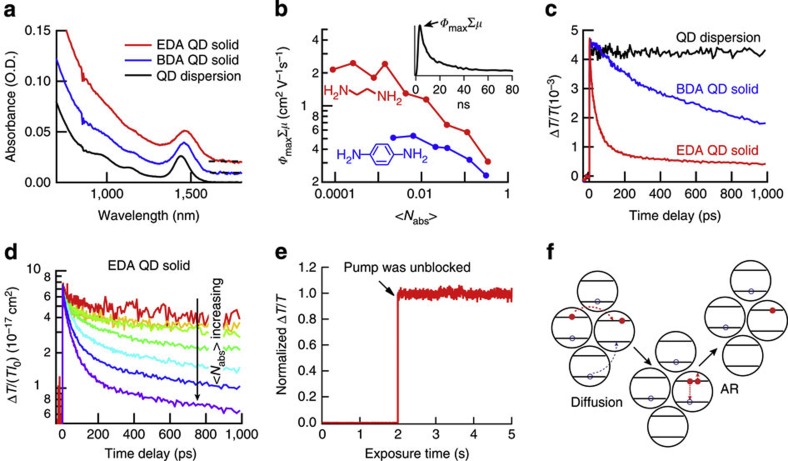
Optoelectronic experiments on PbSe QD solids. (**a**) Absorption spectra of a 4.4-nm PbSe QD dispersion and two QD films with 1,2-ethanediamine (EDA) and 1,4-benzenediamine (BDA) as the capping ligands. The absorption spectra of the QD solids are offset for clarity. (**b**) The product *Φ*_max_Σ*μ* of the charge carrier generation yield and the sum of electron and hole mobilities obtained from TRMC measurements, as a function of the number of absorbed photons per QD. The inset shows a single TRMC transient. (**c**) Absorption transients with <*N*_abs_>=0.15 reveal very different charge carrier decay kinetics in the QD dispersion and the QD solids. All samples are excited at 795 nm and probed at the first exciton maximum. (**d**) The fluence-normalized excitation density-dependent absorption transients in the EDA QD solid, with <*N*_abs_> from 0.0023 to 0.15, indicate a higher-order decay process. (**e**) Real-time absorption transient of a PbSe BDA QD solid at <*N*_abs_>=1. The absorption bleach is recorded at a fixed pump-probe delay of 20 ps as a function of exposure time. The fact that the signal is constant excludes temperature or charging effects on the decay kinetics. (**f**) A schematic of diffusion-mediated AR.

PbSe QD solids were prepared using a layer-by-layer procedure with 1,2-ethanediamine (EDA) or 1,4-benzenediamine (BDA) as the capping ligands to substitute for the much longer original oleylamine ligands. This results in optically smooth films with a mirror-like appearance. Their absorption spectra are shown in Fig. 1a.

The photoconductance of the QD solids was studied with the time-resolved microwave conductance (TRMC) technique^12^,^13^. Figure 1b shows the product *Φ*_max_Σ*μ* of the yield of charge carrier generation *Φ*_max_ (that is, the number of mobile electrons and holes per absorbed photon at the maximum of the transient microwave conductance) and the sum of the electron and hole mobilities Σ*μ* as a function of the average number of photons absorbed per QD, <*N*_abs_>. <*N*_abs_> is obtained as *I*_0_*σ*, where *I*_0_ is the photon fluence in the excitation pulse and *σ* is the absorption cross-section, which is taken from ref. 14, where it was determined for colloidal PbSe QD dispersions. The uncertainty in the fluence is of the order of 10%. However, the absorption cross-section contains an uncertainty of approximately a factor 2, as it will be different in QD films than in dispersions^8^,^15^,^16^. This implies that the uncertainty in <*N*_abs_> is also approximately a factor 2.

For both films, at high excitation density the observed *Φ*_max_Σ*μ* decreases with increasing laser intensity, whereas at low excitation density *Φ*_max_Σ*μ* is independent of <*N*_abs_>. For higher <*N*_abs_>, the magnitude of *Φ*_max_ decreases because of a higher-order charge decay process that takes place within the 3-ns excitation pulse and reduces the maximum photoconductance. The charge carrier mobilities are obtained at the lower limit of excitation density, where *Φ*_max_ is not influenced by higher-order recombination. For the EDA QD solid, we have previously shown that all excitons rapidly dissociate into mobile charge carriers and that (below <*N*_abs_>=10^−3^) *Φ*_max_ is ~1 (ref. 17). Hence, the value of *Φ*_max_Σ*μ* at low intensity is equal to Σ*μ*. The longer BDA ligands lead to a larger separation of QDs compared with EDA ligands, resulting in a smaller electronic coupling and lower charge carrier mobility. From studies of the dependence of conductance on the capping ligand length^18^,^19^, the value of the charge carrier mobility is expected to be 0.6 cm^2^ V^−1^ s^−1^ for the BDA solid, whereas we find *Φ*_max_Σ*μ*=0.5 cm^2^V^−1^ s^−1^ in the measurements. This implies that *Φ*_max_ is also ~1 in the BDA QD solid.

### TA studies

To investigate the higher-order recombination on a sub-nanosecond timescale, we applied femtosecond TA spectroscopy. Charge carriers were detected exploiting the fact that the bleach of the 1S_h_1S_e_ interband transition is proportional to the charge carrier density^20^,^21^,^22^,^23^. Figure 1c shows transients of the interband absorption bleach for a QD dispersion and for the two films excited with 795 nm pump pulses at an excitation density of <*N*_abs_>=0.15. In the QD dispersion, there is no appreciable decay of the 1S_h_1S_e_ bleach up to 1 ns, whereas in the two films the bleach decays with very different decay rates.

For isolated dispersed QDs, three processes usually dominate the decay of excitons: radiative recombination, AR and surface trapping. Radiative combination occurs on a microsecond timescale in PbSe QDs^24^,^25^; hence, it is absent in the 1-ns time window of Fig. 1c. For QDs in dispersion, AR requires at least two excitons. The probability of having multiple excitations per QDs follows a Poisson distribution:


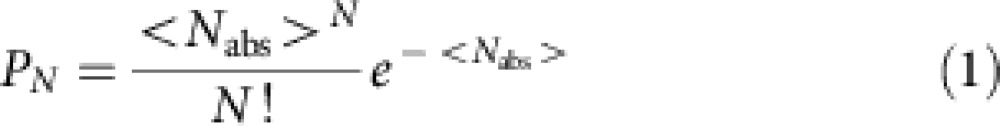


where *P*_*N*_ is the probability of having *N* excitations on a QD. According to equation (1), only 1% of all QDs carry a biexciton at <*N*_abs_>=0.15. Hence, AR is negligible for the dispersion measurement in Fig. 1c. Finally, the absence of decay indicates that no trapping occurs on this timescale.

Figure 1c shows that in the QD solids, a significant decay of charge carriers occurs within 1 ns. In addition, it shows that the decay is faster in the EDA film than that in the BDA film. Figure 1d shows TA transients for the EDA film normalized by the photon fluence in the laser pulse *I*_0_, with <*N*_abs_> varying from 0.0023 to 0.15. Clearly, the decay gets faster as the charge carrier density is increased. At low fluence, decay is almost absent within 1 ns. A similar observation is made for the BDA film, albeit with overall much slower decay (see Supplementary Fig. S1). We note that the observed decay in the TA experiments quantitatively matches the reduction of the *Φ*_max_Σ*μ* value at high fluence in the TRMC experiments. This is true for both EDA and BDA samples.

We attribute the higher-order decay observed here to AR. In QD dispersions, AR only occurs if the number of excitons per QD exceeds one, as the AR process requires at least a trion: two electrons and a hole (*eeh*) or two holes and an electron (*ehh*). Therefore, it is surprising at first sight that AR in QD solids is already active at <*N*_abs_>=0.001. One possible explanation could be that charge trapping results in long-lived charges that survive longer than the time between two pump pulses, which is 2 ms for these experiments. This could result in a built-up of charge density giving rise to AR.

To investigate this scenario, we have performed ‘real-time’ TA experiments: we monitored the TA signal at fixed 20 ps pump-probe time delay as a function of exposure time after the pump is unblocked. A representative measurement is shown in Fig. 1e. Note that an absorption transient was not recorded, that is, the pump-probe delay was not changed. Rather the absorption bleach at fixed pump-probe delay was monitored on a timescale of seconds to test for possible effects of charging or heating. The pump intensity corresponds to <*N*_abs_>=1. If charging takes place and results in additional AR, the decay should become faster and the signal at 20 ps delay should decrease with exposure time. The fact that the signal remains perfectly constant shows that charging does not influence the decay kinetics in these measurements.

We cannot exclude that some photo charging takes place, but we conclude that it does not affect the absorption transients shown in Fig. 1c,d. Additional evidence for this is presented as Supplementary Fig. S2. This implies that the density of mobile charge carriers that is left after 1 ms (when the next probe pulse arrives) is much lower than the density of charges that is generated by the laser pulse itself.

Although at low excitation density Auger reactive species are not photogenerated directly, they could form via charge carrier diffusion, as illustrated in Fig. 1f, leading to AR at <*N*_abs_><<1. The hopping rate of charge carriers may be estimated from the measured mobility via the Einstein–Smoluchowski relation 
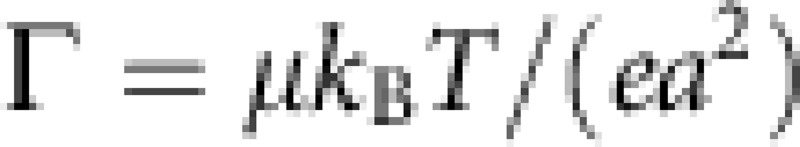
, where *e* is the elementary charge, *a* is the hopping distance, *k*_B_ is Boltzmann’s constant and *T* is the temperature. This gives the rate with which a charge carrier hops to a specific neighbour site^26^,^27^. The total rate of hopping out of a QD is greater by the number of neighbours in the film and is (0.4 ps)^−1^ for the EDA QD solid and (1.6 ps)^−1^ for the BDA QD solid (assuming 12 nearest neighbours, a mobility of 2 cm^2^ V^−1^ s^−1^ and an interparticle distance of 4.7 nm). Therefore, diffusion of charge carriers could result in AR on a picosecond timescale. Charge carrier diffusion-mediated AR can also explain why the decay is faster for EDA ligands than for BDA ligands, as the charge carrier mobility is significantly higher for the EDA QD solid.

The first Auger reactive species that can be formed via diffusion is a trion. As a starting point, we could assume that AR is due to trions only. The expected trion lifetime for this size of PbSe QDs is 70 ps^28^, whereas the half lifetime of the TA decay at <*N*_abs_>=0.15 is only 25 ps. Hence, it is unlikely that trions are responsible for the observed decay.

The scaling of the AR rate with the number of charge carriers has been investigated for QDs in dispersion^28^,^29^. For our PbSe QD solids we adapt the expression from ref. 28 as follows:


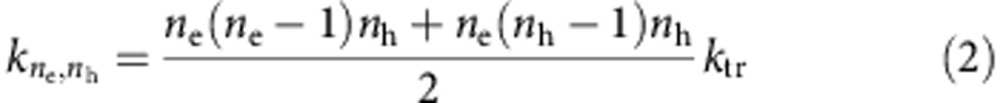


where *n*_e_ and *n*_h_ are the number of electrons and holes in a QD, respectively, 
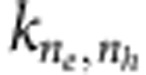
 is the AR rate of (*n*_e,_*n*_h_)→(*n*_e_–1,*n*_h_–1) and *k*_tr_ is the trion AR rate. For an experimentally determined biexciton decay rate of (17.5 ps)^−1^ (ref. 22), equation (2) predicts a quadraexciton (*n*_e_=4,*n*_h_=4) decay rate of (1.5 ps)^−1^. Such highly charged QDs could in principle explain the observed fast decay, if they would form.

At an excitation density of 0.15 excitations per QD, the probability of directly photoexciting QDs multiple times is negligible. Ultrafast dissociation of excitons and diffusion of charge carriers may generate charged QDs with various combinations of electrons and holes. If these charge carriers reach equilibrium and are distributed with equal probability over all QDs in the film, this distribution would again be Poissonian. In this case, the fraction of QDs that contain a trion is only 0.15%. (The total charge density *n*_tot_, containing both electrons and holes, is equal to 2<*N*_abs_>. The probability that three charges occupy the same QD is then given by *P*_3_=*n*_tot_^3^exp(−*n*_tot_)/3!, which equals 0.3% at <*N*_abs_>=0.15. Of these QDs with three charges, only half carry trions, the other half carry three electrons or three holes.) Therefore, no significant AR is expected.

However, the above situation oversimplifies the system as it assumes all QDs are identical and neglects Coulomb interactions between charges. Significant energy disorder exists in QD solids, as evidenced, for instance, by a relatively broad first exciton absorption peak. In the EDA film, the energy disorder *w* is about 50 meV, estimated from the Gaussian width of the 1S absorption peak. We have shown recently that charge carriers undergo fast thermalization after initial photoexcitation and are more likely to occupy sites with lower energy^27^. As a result, charge carriers congregate in low-energy sites forming QDs with a higher number of charge carriers where AR is more efficient. To investigate the effect of this carrier congregation in low-energy sites, we have performed Monte Carlo simulations.

### Monte Carlo simulations

We use a three-dimensional cubic lattice with periodic boundary conditions. Electrons and holes diffuse via nearest neighbour hopping according to the Miller–Abrahams rate as^30^:


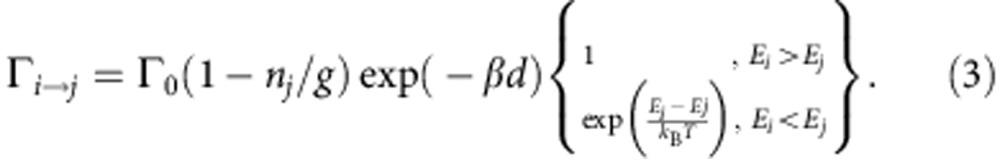


where 
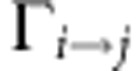
 is the hopping rate from site *i* to *j*, 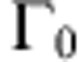 is an attempt hopping rate, *n*_*j*_ is the number of electrons (or holes) in site *j*, *g* is the 1S level degeneracy (*g*=8 for PbSe QDs^20^,^27^,^31^), *β* is the tunnelling decay parameter with value of 0.87 Å^−1^ as determined previously^19^, *d* is the edge-to-edge interparticle spacing, *E*_*i*_ and *E*_*j*_ are the site energies, *k*_B_ is Boltzmann’s constant and *T* is the temperature of 298 K. The factor (1−*n*_*j*_*/g*) is included to take into account that charge carriers can only hop into empty levels. Charge carriers residing within the same QD decay via AR with decay rates following equation (2).

Electron–hole pairs are initially distributed on the lattice according to a Poisson distribution (equation (1)) to simulate photoexcitation of (multiple) excitons. Energy disorder, positional disorder and Coulomb interactions between charges are included in the simulations as described in detail in the Supplementary Methods. Briefly, the energy disorder of typically 50 meV is derived from the width of the 1S absorption peak. Coulomb interactions between charges are estimated using the work of Delerue^32^. A typical electron–hole Coulomb interaction is 80 meV (see Supplementary Note 1 for a derivation and discussion of this value). Positional disorder is taken into account in a crude way by assuming that the interparticle spacing *d* is a uniform distribution between 0 and typically 1.0 nm.

**Figure 2:**
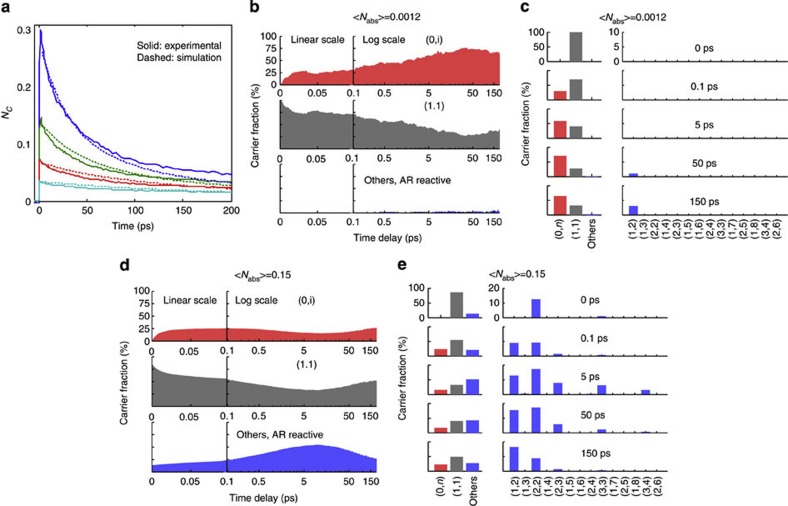
Monte Carlo simulations of AR in QD solids. (**a**) Simulated (dashed line) and experimental (solid line) carrier decay traces with initial excitation density <*N*_abs_> from 0.15 to 0.019. *N*_c_=2<*N*_abs_> is the total charge carrier density (electrons plus holes). (**b**,**d**) Charge carrier combinations (0,i; red shaded area), (1,1; grey shaded area) and others (AR reactive; blue shaded area) with (**b**) <*N*_abs_>=0.0012 and (**d**) <*N*_abs_>=0.15 on a lin-log timescale (the first 100 fs are on a linear timescale; subsequently a logarithmic scale is used). (**c**,**e**) Histograms of the MCDs for five delay times with (**c**) <*N*_abs_>=0.0012 and (**e**) <*N*_abs_>=0.15. The same colour coding is used as in **b** and **d**.

Using our Monte Carlo simulations, we can reproduce the experimental data, as shown in Fig. 2a, with a trion lifetime of 71 ps and a charge carrier mobility of 1.5 cm^2^ V^−1^ s^−1^. The trion lifetime matches the expected lifetime of 70 ps^28^; the carrier mobility from the simulations agrees with the experimentally determined mobility. We need to include both energy disorder and positional disorder to fit the data with the experimentally determined mobility. Omitting positional disorder results in a mobility that is approximately ten times higher (see Supplementary Note 2). Similar results are obtained for QDs with BDA ligands, in which case both the experimental and simulated mobilities are lower (Supplementary Fig. S3) and for smaller QDS with EDA ligands, in which case the experimental and simulated mobility are somewhat higher (Supplementary Fig. S4). As especially the modelling of positional disorder in our simulation is rather approximate, the match between the experimental and simulated mobility for the QD solid with EDA ligands must be considered coincidental to some extent.

In Fig. 2, we show the fraction of charge carriers in various carrier combinations as a function of time. (*n*_1_,*n*_2_) represents the charge carrier combination of *n*_1_ electrons and *n*_2_ holes or *vice versa*. We distinguish groups of charge carriers consisting of only electrons or holes (0,*i*), excitons (1,1) and other combinations of electrons and holes (where AR could occur; hence referred to as ‘AR reactive’), with red, grey and blue colours, respectively. The fractions of AR-reactive charge carriers are also shown in histograms termed multiple carrier distributions (MCDs) for several delay times, Fig. 2c,e.

At zero time delay, (multiple) excitons are formed following a Poisson distribution. As a result of the high hopping rate, these excitons rapidly dissociate and the charges redistribute over the various carrier combinations. First, we focus on the situation where the excitation density is very low and little AR occurs. This situation, with <*N*_abs_>=0.0012, is shown in Fig. 2b,c. Figure 2b shows that the fraction of excitons rapidly decreases within 50 fs, and that at the same time the fraction of (0,*i*) increases up to ~25%. On a longer timescale of ~30 ps the fraction of free charges increases further to ~80%.

The initial, rapid exciton dissociation can be understood by considering that as a result of energy disorder, excitons on high-energy sites may dissociate into charges at low-energy sites without significant increase in energy. A similar explanation was recently given for the dissociation of charge transfer complexes in disordered organic heterointerfaces^33^. The 1.5-cm^2^ V^−1^ s^−1^ carrier mobility used in these simulations corresponds to a (470-fs)^−1^ hopping rate. However, this is the hopping rate after thermalization of the charge carriers. From our simulations, we determine that the average hopping rate is 30 times higher before thermalization. Therefore, it can be understood that the simulated initial exciton dissociation occurs on a <50-fs timescale. Further exciton dissociation takes longer as the remaining excitons have a lower energy. This implies that exciton dissociation will be slowed down by both disorder and electron–hole attraction. Even so, exciton dissociation is rapid and nearly complete in 30 ps. We remark that, in our simulations, electrons and holes do not form strongly bound excitons but are in dynamic equilibrium with single charges. The probability of forming an exciton from free charges depends on the square of the charge density, and therefore the fraction of free charges is a function of charge density.

Figures 2d,e show simulations for a higher excitation density. In this case, after the initial exciton dissociation, charge carriers diffuse through the film, congregate at lower-energy sites and form hot spots for AR (blue area in Fig. 2d; blue bars in Fig. 2e). The fraction of Auger reactive species in the film increases up to ~10 ps delay time. The MCDs in Fig. 2e clearly show that besides trions there are significant contributions from QDs with more charge carriers where AR is more efficient (equation (2)); this explains the fast charge carrier decay. As time increases, AR takes place and the overall charge carrier density drops, the MCD shifts to less highly charged species and the overall fraction of Auger reactive species decreases, resulting in slower decay. The above simulations reveal that the fast carrier decay is due to an enhanced AR as a result of carrier congregation in low-energy sites.

## Discussion

The half lifetime of the Auger decay may be extracted from Fig. 1d and varies from 25 ps at 0.15 absorbed photons per QD to 550 ps at <*N*_abs_>=0.01, for the EDA-treated PbSe QD solid investigated here. These carrier densities are high compared with the charge carrier densities in QD solar cells and LEDs under operational conditions. The charge density in a QD solar cell with a charge carrier mobility of 1 cm^2^ V^−1^ s^−1^ can be estimated to be ~10^−6^ charges per QD, whereas the residence time of charges in the solar cell is ~5 ns (Supplementary Note 3). An extrapolation of the Auger half lifetime (Supplementary Fig. S5) shows that at such a charge density, AR is negligible within the residence time of the charges. Hence, the AR observed here is no issue of great concern for QD solar cells, unless they operate under concentrated sunlight. A similar reasoning can be made for QD LEDs, which typically operate at a charge density of 10^−4^ charges per QD. At this charge density, the extrapolated Auger half lifetime is 80 ns, which should be compared with the radiative lifetime, which is of the order of a microsecond for lead chalcogenide nanocrystals^25^. This implies that AR may affect the efficiency of Pb chalcogenide QD LEDs. A recent paper by Sun *et al.*^11^ demonstrated that the efficiency of PbS QD LEDs may be optimized by increasing the length of the ligands. This results in a reduction of the charge carrier mobilities and a concomitant reduction of AR.

In addition to posing a threat to certain optoelectronic devices, the fast AR observed here could also be exploited for fast all-optical switching in the NIR. For such a switch, a pump-induced change in transmission should occur at relevant telecom wavelengths (1.3 or 1.5 μm), with a high modulation and a fast return to the ground state^34^. The observed AR results in a 1S_h_1S_e_ absorption bleach that returns to ~0.1 of its maximum in ~100 ps. This enables a 10-GHz optical switch with a modulation of 10. The modulation and switching speed may be improved by using fast carrier diffusion as a design principle.

**Figure 3:**
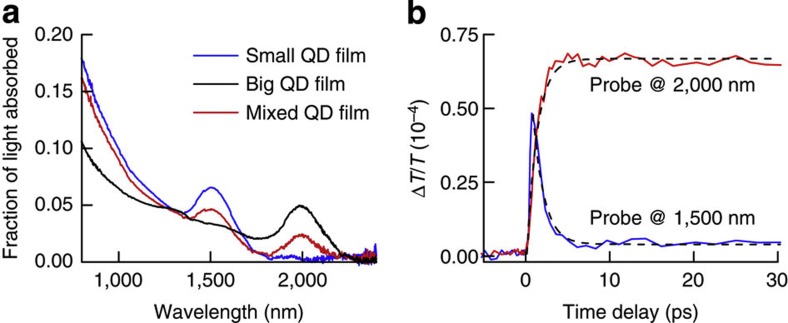
Demonstration of fast carrier congregation. (**a**) Absorption spectra of QD films that consist of purely 4.8 nm QDs (small QD film, blue line), purely 7.5 nm QDs (big QD film, black line) or alternating layers of these small and large QDs (mixed QD film, red line). (**b**) TA spectra for the mixed QD film shown in **a**, excited at 795 nm with <*N*_abs_>=0.0024 and probed at 1,500 and 2,000 nm. The decay of the TA signal at 1,500 nm and the concomitant increase at 2,000 nm reveal charge carrier transfer from the small QDs to the large ones. Dashed lines are exponential fits to the data.

Figure 3 shows TA measurements on a QD film that is composed of two sizes of QDs with diameters of 4.8 and 7.5 nm. Upon photoexcitation with 795 nm pump pulses, the 1S_h_1S_e_ absorption bleach of the smallest QDs exhibits a fast modulation. As charges diffuse from the small to the big QDs, the signal at 1.5 μm exhibits an exponential decay with a time constant of 1.3 ps (dashed line in Fig. 3b). A film consisting of purely small QDs does not show appreciable decay under these conditions (see Supplementary Fig. S6). At the same time, the absorption bleach at the first exciton maximum of the big QDs grows in with the same time constant. This allows for an ~0.8-THz optical switch. We note that such fast transfer rates are only obtained if phase separation of small and big QDs is carefully avoided (see Supplementary Fig. S7). The same concept of carrier congregation in large QDs could possibly be used to create population inversion in low threshold NIR lasers. Instead of direct intense pumping, a large excess of small QDs could act as antennas and transfer the generated charge carriers to a few large QDs in the film.

In summary, AR was found to be a very efficient charge carrier decay path in PbSe QD solids. Monte Carlo simulations reveal that energy disorder leads to efficient charge carrier congregation in low-energy sites, where fast AR occurs, even at low average carrier density. This disorder-enhanced AR is not specific for QD solids but is expected to occur in all disordered materials. We estimate that AR is not a significant problem for QD solar cells but that it does affect the efficiency of QD LEDs. Efficient congregation of charge carriers may be exploited to achieve optical gain and fast optical switching in the NIR.

## Methods

### QD film preparation and optical characterization

PbSe QDs were synthesized following ref. 35. Oleylamine (14 ml) and 0.38 g PbCl_2_ were degassed under vacuum at 100 °C, followed by heating to 160 °C under nitrogen. Next, 260 ml Sn[N(SiMe_3_)_2_]_2_ dissolved in 2 ml of trioctylphosphine and 6 ml of 1 M Se in trioctylphosphine were mixed in a syringe and immediately injected into the PbCl_2_ oleylamine solution. The reaction mixture was kept at the temperature after the injection for 2 min and cooled with a water bath. The resulting solution was filtered with a 200-nm pore size filter to remove residual PbCl_2_. The QDs were precipitated with butanol and finally dried under vacuum. The QDs were dispersed in tetrachloroethylene for absorption measurements and in hexane for dip coating.

Quartz substrates were silanized with (3-aminopropyl) triethoxysilane^36^. PbSe QD solids were produced by a mechanical dip coater (DC Multi-8, Nima Technology) mounted inside a nitrogen glove box. A silanized quartz substrate was dipped alternately into a 10-μM solution of PbSe QDs in hexane for 60 s, a 0.1-M solution of replacing ligands in methanol for 60 s and a rinsing solution of methanol for 30 s. The dipping procedure was repeated 15 times. EDA or BDA were used as the capping ligands to substitute for the much longer original oleylamine ligands. This results in optically smooth films with a mirror-like appearance. For the film with mixed QD sizes, 10 layers of 4.8 nm PbSe QDs and 10 layers of 7.5 nm QDs were deposited in an alternating sequence using the same layer-by-layer procedure described above using EDA as replacement ligands.

Optical absorption spectra were recorded with a Perkin–Elmer Lambda 900 spectrometer equipped with an integrating sphere. The spectra were corrected for scattering and reflection.

### TRMC experiments

The photoconductance of QD solids was investigated using the TRMC technique^12^,^13^,^37^. Photoexcitation laser pulses with a duration of 3 ns were obtained by pumping an optical parametric oscillator with the third harmonic of a Q-switched Nd:YAG laser (Opotek Vibrant 355 II). The sample was excited at 1.8 eV. The photon fluence *I*_0_ was varied between 1 × 10^11^ and 1 × 10^14^ photons per cm^2^ per pulse. Upon photoexcitation, the change in microwave power reflected was measured. For small photo-induced changes in the real conductance of the sample, Δ*G*(*t*), and negligible change in imaginary conductance, the relative change in microwave power can be written as Δ*P*(*t*)/*P*=−*K*Δ*G*(*t*) , where *K* is a sensitivity factor which has been determined previously. The photoconductance Δ*G*(*t*) can be expressed as Δ*G*(*t*)=*eβI*_0_*F*_a_*Φ*(*t*)Σ*μ*, where *e* is the elementary charge, *β* is the ratio between the broad and narrow inner dimensions of the waveguide, *I*_0_ is the photon fluence in the laser pulse, *F*_a_ is the fraction of light absorbed by the sample, *Φ*(*t*) is the number of mobile charge carriers at time *t* per absorbed photon and Σ*μ* is the sum of the electron and hole mobilities. The time resolution of the TRMC set-up is limited by the 3-ns laser pulse.

### TA experiments

The samples were excited and monitored by pump and probe pulses from a chirped-pulse amplified laser system (Mira-Legend USP, Coherent Inc.), running at 1 kHz and delivering pulses of 60 fs, 2.2 mJ and at a wavelength of 795 nm. Tunable infrared and visible pulses (<100 fs) were generated by optical parametric amplification (Topas-800-fs and Opera, Coherent Inc.). Pump and probe beams were imaged onto InGaAs pin photodiodes (Hamamatsu G5853-23, G8605-23). The two beams were spatially separated downstream of the sample. Orthogonal polarization of the beams allowed further separation by means of a polarizer.

## Author contributions

Y.G. and C.S.S.S. synthesized the quantum dots, prepared the QD films and performed TA experiments. Y.G. performed the TRMC experiments and the Monte Carlo simulations; A.J.H. and Y.G. designed the experiment and wrote the paper; Y.G., A.J.H., J.M.S. and L.D.A.S. analysed the data.

## Additional information

**How to cite this article:** Gao, Y. *et al.* Disorder strongly enhances Auger recombination in conductive quantum dot solids. *Nat. Commun.* 4:2329 doi: 10.1038/ncomms3329 (2013).

## Supplementary Material

Supplementary Information Supplementary Figures S1-S10, Supplementary Tables S1-S2, Supplementary Notes 1-3, Supplementary Methods and Supplementary References
