# Supplementary material for: Disorder strongly enhances Auger recombination in conductive quantum-dot solids
Source: Nat Commun. 2013 Sep 13;4:2329. doi: 10.1038/ncomms3329 (PMC3778520; doi:10.1038/ncomms3329)
Supplement: Supplementary Information — Supplementary Figures S1-S10, Supplementary Tables S1-S2, Supplementary Notes 1-3, Supplementary Methods and Supplementary References [file ncomms3329-s1.pdf]

Supplementary Figures

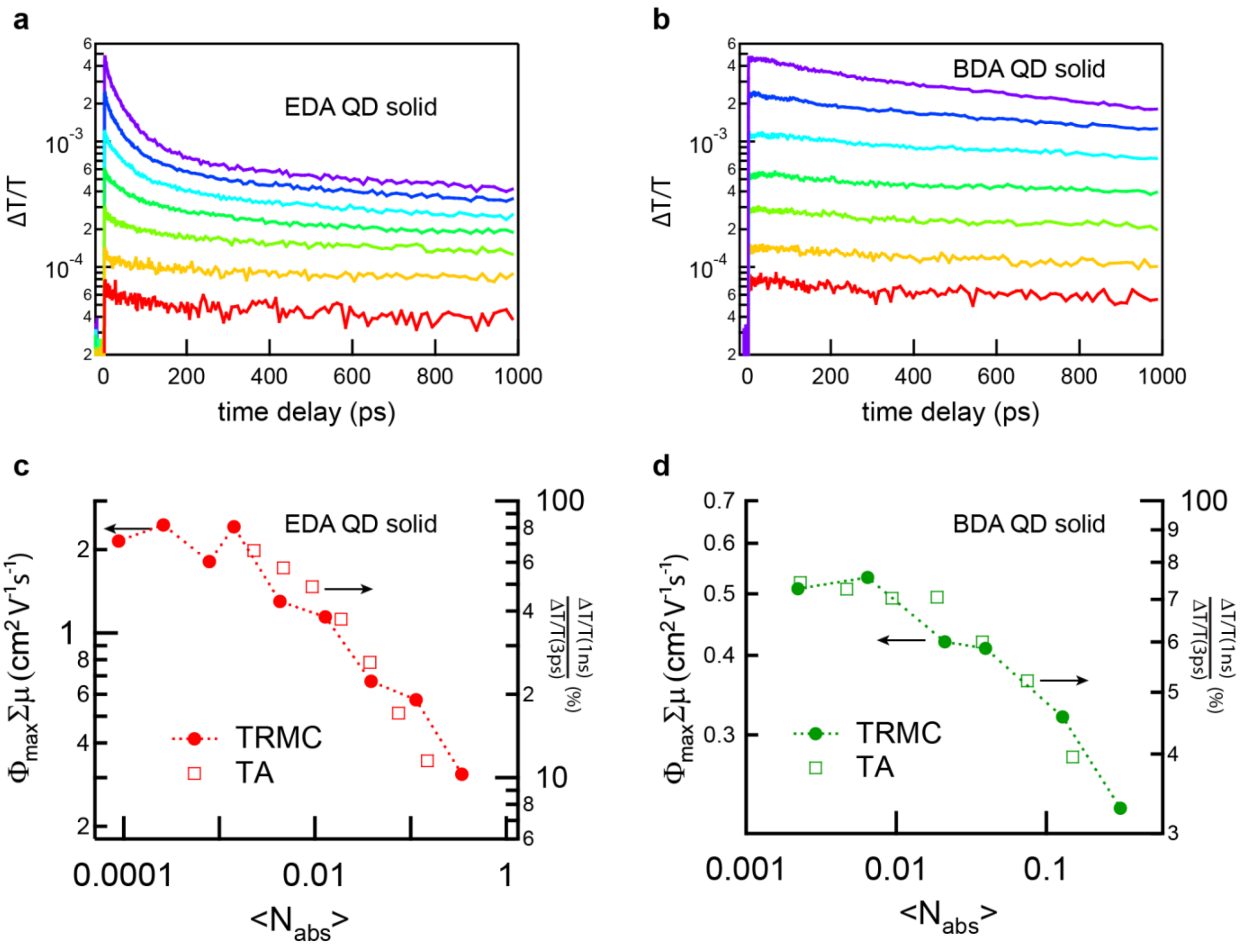

Supplementary Figure S1 Consistence between TRMC and TA results. a, b, Excitation density dependent transient absorption traces for EDA and BDA QD solids. From these traces, the ratio of the bleach at 1 ns to 3 ps (peak value) is determined. **c, d**, The $\Phi_{max}\Sigma\mu$ value determined by TRMC (dots) and the ratio of transient absorption bleach at 1 ns and at 3 ps (peak value) (open squares) show identical $< N_{abs} >$ dependence. An excellent agreement between them is observed. The fact that the TRMC photoconductivity at 3 ns and the TA at 1 ns have such a similar intensity dependence shows that the photoconductivity is due to same excitations that result in the $1S_e1S_h$ absorption bleach, hence due to electrons and holes in the $1S_e$ and $1S_h$ levels. The identical excitation density dependence observed here, along with the fact that the traces of THz absorption by mobile charge carriers are identical to TA traces,[1] suggests that the yield η of photo-generation of mobile charge carriers is close to unity for both EDA and BDA QD solids. As both the THz absorption and the microwave absorption are due to mobile charge carriers, identical decay kinetics of THz absorption and TA shows that the decay on sub-nanosecond is due to decay of mobile charge carriers, while the identical excitation dependence of $\Phi_{max}\Sigma\mu$ and the TA ratio shows that what remains from the fast decay are also mobile charge carriers.

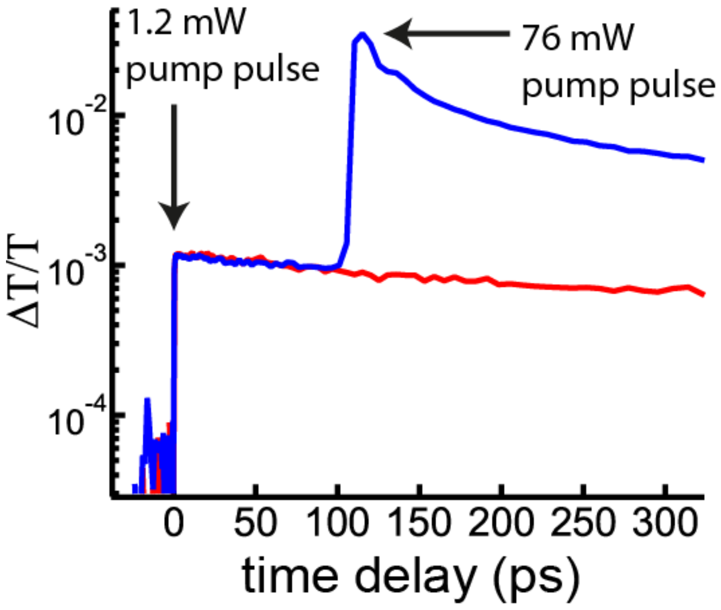

Supplementary Figure S2 Absence of charging effects on the observed transients. We performed a pump-probe experiment on a BDA QD film with 795 nm, 1.2 mW pump pulses, corresponding to $\langle N_{abs} \rangle = 0.09$. The resulting transient, probed at the first exciton maximum, is shown as the red line in the figure. Subsequently we performed the same experiment but we introduced a second, high-power, pump pulse 100 ps after the first (blue line). The power of the second pump pulse corresponds to $\langle N_{abs} \rangle = 5.7$, i.e. much higher than in all other experiments discussed here. The pump-probe and pump-pump-probe transients are identical in the first 100 ps. This demonstrates that the observed transient absorption signals are purely due to the weak pump pulse at zero time delay. Hence, any eventual long-term increase in charge density or temperature has no detectable effect on the decay kinetics. This implies that the density of mobile charge carriers that is left after 1 ms (when the next laser pulse hits the sample) is much lower than the density of charges that is generated by the laser pulse itself.

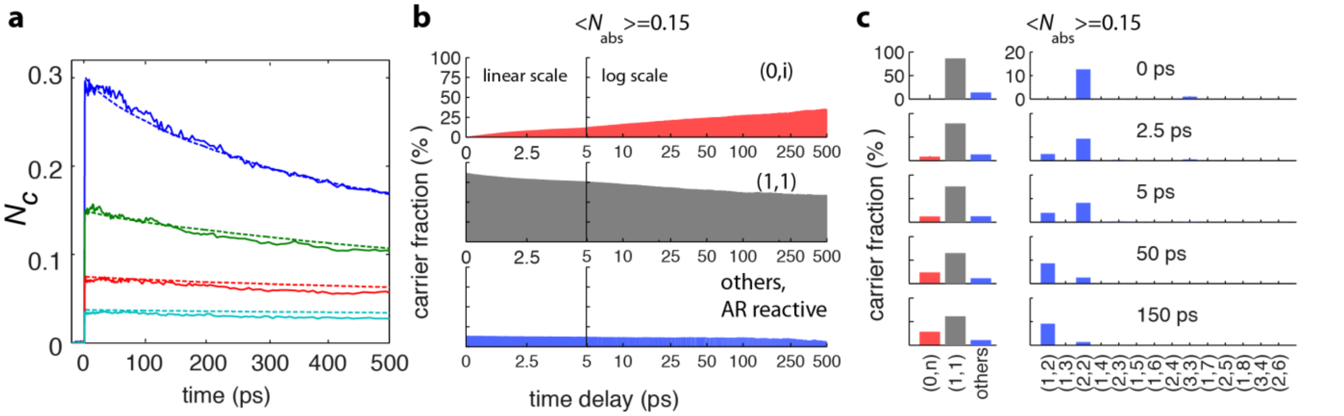

Supplementary Figure S3 Experimental results and simulations for BDA QD solids. a, Experimental (solid lines, TA) and simulated (dashed lines, Monte Carlo simulations**)** carrier decay traces for a QD solid of 4.5 nm PbSe QDs and BDA ligands at various excitation densities. **b,** Charge carrier combinations (0,i), (1,1) and others (AR reactive) with $< N_{abs} > = 0.15$ on a lin-log time scale (the first 100 fs are on a linear time scale, subsequently a logarithmic scale is used). **c,** Histograms of the Multiple Carrier Distributions for five delay times. The parameters used for the Monte Carlo simulations were as follows: Electron-hole Coulomb interaction energy 80 meV, energy disorder 36 meV, positional disorder 1 nm, a mobility of 0.015 cm^2/Vs and a trion decay rate of 70 ps. Compared to the EDA QD solids discussed in the main text the energy disorder is somewhat smaller (since the 1S peak in the absorption spectrum is narrower) and the mobility is lower. The lower mobility results in slower exciton dissociation and carrier diffusion and, hence, slower Auger recombination.

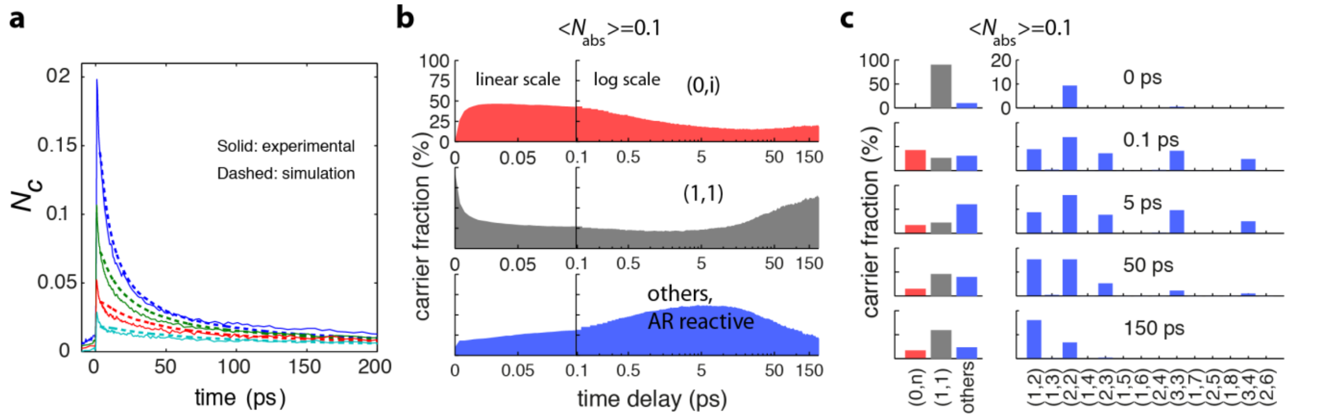

Supplementary Figure S4 Transient absorption results and Monte Carlo Simulations for a second QD size a, Experimental (solid lines, TA) and simulated (dashed lines, Monte Carlo simulations**)** carrier decay traces for a QD solid of 3.8 nm PbSe QDs and EDA ligands at various excitation densities. **b,** Charge carrier combinations (0,i), (1,1) and others (AR reactive) with $< N_{abs} > = 0.15$ on a lin-log time scale (the first 100 fs are on a linear time scale, subsequently a logarithmic scale is used). **c** Histograms of the Multiple Carrier Distributions for five delay times. For these smaller QDs the trion lifetime is shorter[2], 60 ps. The other simulation parameters were as follows: electron-hole Coulomb energy 98 meV, energy disorder 90 meV, positional disorder 1 nm and a mobility of 12.9 cm^2/Vs. The higher mobility and shorter trion lifetime result in Auger recombination that is even faster than for the 4.4 nm PbSe QD solid discussed in the main text.

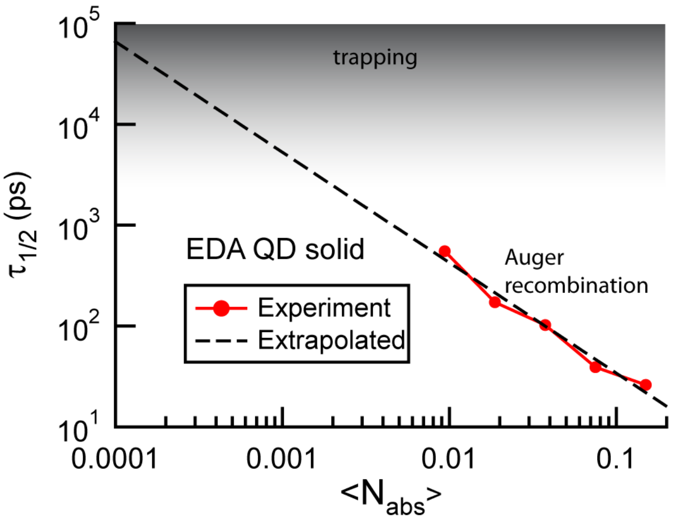

Supplementary Figure S5: Half lifetime of the Auger decay. The half lifetime of the Auger decay is extracted from Figure 1d in the main text for various excitation densities. The data are shown on a log-log scale and are extrapolated to obtain a rough estimate of the half lifetime at low excitation density.

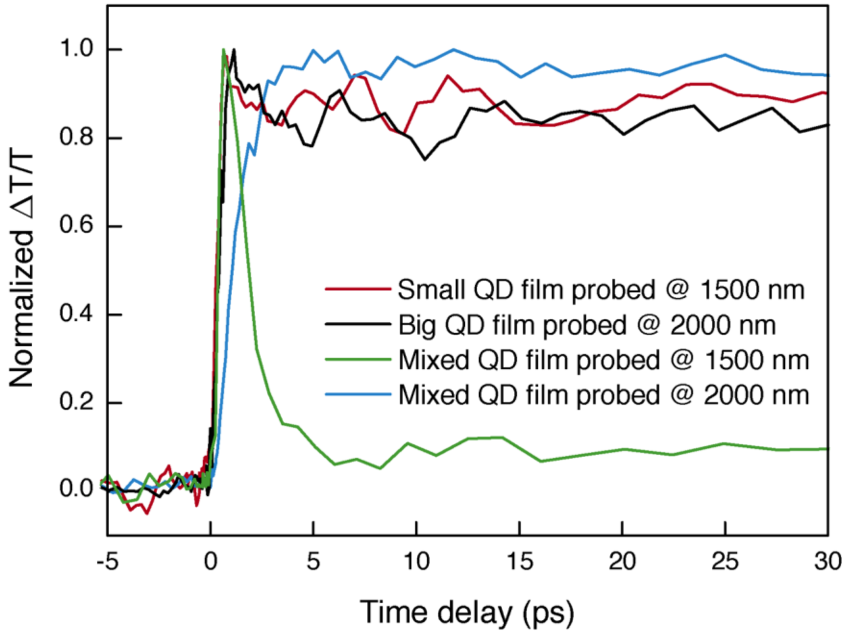

Supplementary Figure S6 Additional transients form mixed QD films. Absorption transients on QD films that consist of a purely 4.8 nm QDs ("Small QD film", red line), purely 7.5 nm QDs ("Big QD film", black line), or alternating layers of these small and large QDs ("Mixed QD film") probed at the 1S exciton maximum of the small QDs (green line) or the big QDs (blue line). The samples are excited with 795 nm pump pulses at an excitation density of 0.0024 per QD, and are probed at the indicated wavelengths. In the mixed QD film a fast decay of the absorption bleach due to the small QDs is accompanied by a similarly fast increase of the absorption bleach of the big QDs.

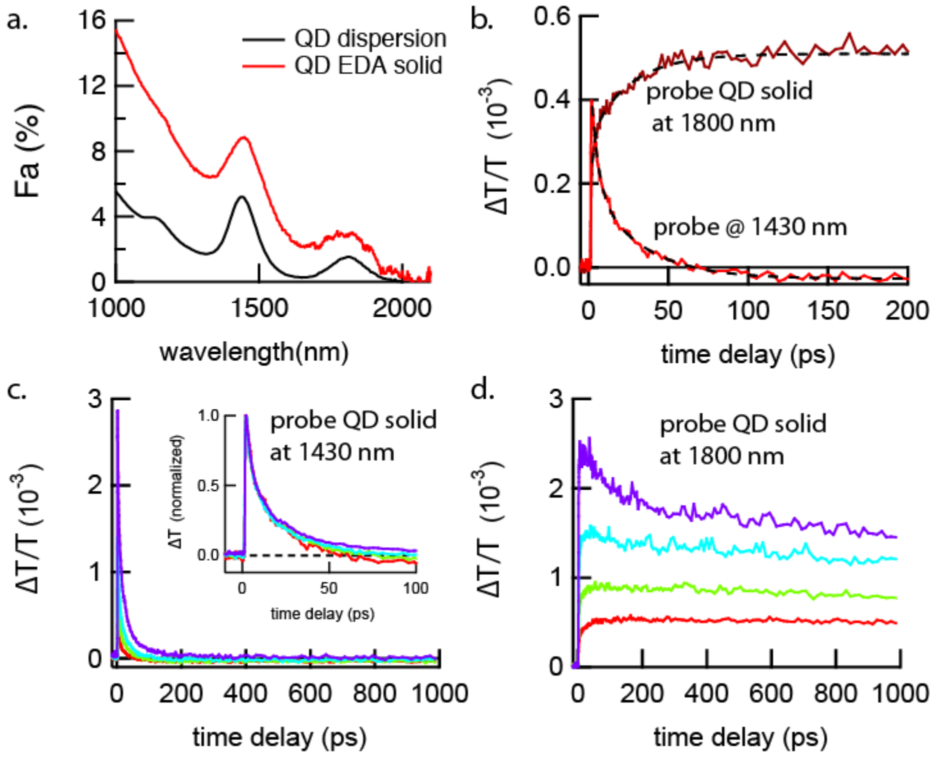

Supplementary Figure S7 Charge transfer in mixed QD films that exhibit phase separation. a, Absorption spectra of a QD dispersion and a film, with two sizes of QDs mixed. Unlike the sample shown in Fig. 3 of the main text, this film was prepared by mixing two QD dispersions and using that mixture for dipcoating. The size ratio of the particles used corresponds to 0.6875. For this size ratio it is both predicted theoretically[3] and observed experimentally for colloidal QDs[4] that phase separation into domains of single component small and large particles occurs and that no mixing takes place. This phase separation leads to lower transfer rates than those shown in Figure 3: $(25\ \text{ps})^{-1}$ here vs. $(1.3\ \text{ps})^{-1}$ in Figure 3, where phase separation is avoided. **b**, Transient absorption spectra with $< N_{abs} >$ of 0.019 probed at 1430 nm and 1800 nm reveal charge carrier transfer from the small QDs to the large ones. **c**, **d**, The excitation density dependent TA spectra in the solid, with $< N_{abs} >$ from 0.019 to 0.15, probed at 1430 nm and 1800 nm indicate that charge carriers first undergo transfer from the small QD to the large ones and then decay via Auger recombination in the big QDs.

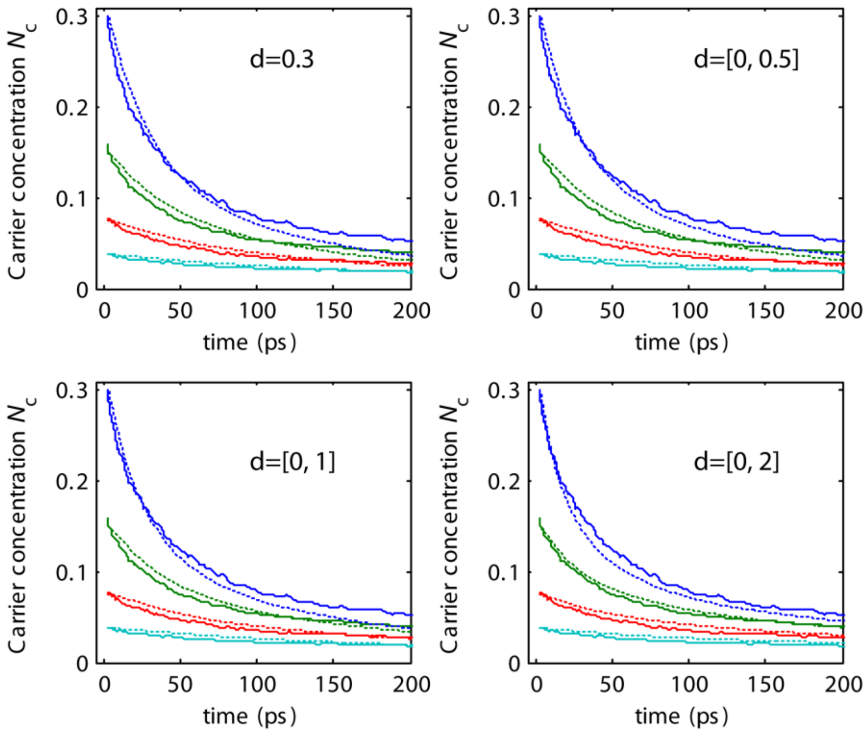

Supplementary Figure S8: Simulated (dashed lines) and experimental (solid lines) charge carrier decay traces. The simulated curves were obtained with varying extent of disorder in inter-particle distance.

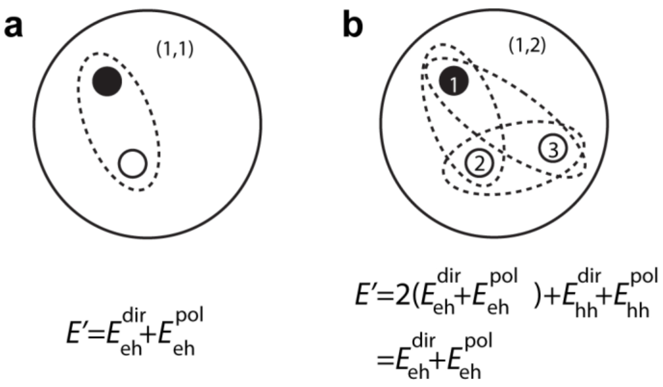

Supplementary Figure S9: A Schematic illustration of how E', *i.e.* the sum of direct Coulomb interaction energy E^{dir} and the cross-polarization energy E^{pol}, is calculated. **a**, E' for an exciton. **b**, E' for a trion (*ehh*) including contributions from interactions between particles 1&2, 1&3, and 2&3.

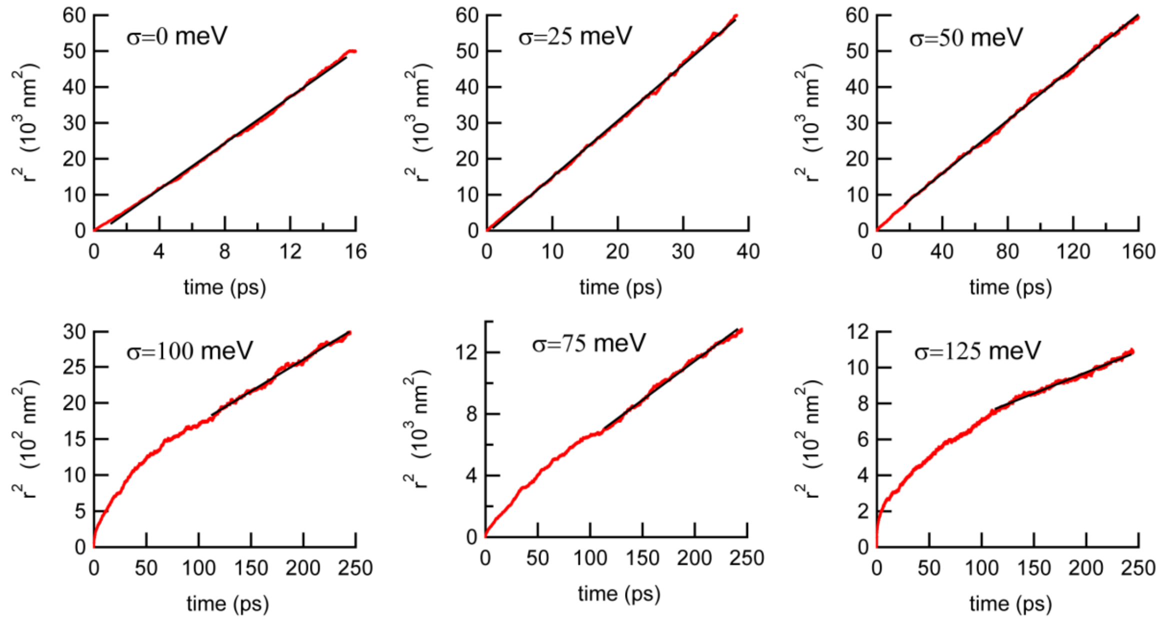

Supplementary Figure S10: The mean squared displacement $\langle r^2 \rangle$ as a function of time for various values of the energy disorder w. Black lines are linear fits to the linear part of the diffusion curves. These fits are used to derive the charge carrier mobility.

Supplementary Tables

Supplementary Table S1 The Coulomb energy change $\Delta E_{\text{Coulomb}}$ is calculated for a carrier hopping between various combinations of charge carriers. A(*n,m*)B(*n,m*) represents two QDs with n electrons and m holes. For clarity only $E_{\text{e,h}}^{\text{dir}}$ and $E_{\text{e,h}}^{\text{pol}}$ are used.

Initial occupation	Final occupation	E'_{initial}	E'_{final}	$\Delta E_{\text{Coulomb}}$
A(0,1)B(0,0)	A(0,0)B(0,1)	0	0	0
A(1,1)B(0,0)	A(1,0)B(0,1)	$E_{\text{e,h}}^{\text{dir}}+E_{\text{e,h}}^{\text{pol}}$	0	$-(E_{\text{e,h}}^{\text{dir}}+E_{\text{e,h}}^{\text{pol}})$
A(2,0)B(0,0)	A(1,0)B(1,0)	$-(E_{\text{e,h}}^{\text{dir}}+E_{\text{e,h}}^{\text{pol}})$	0	$E_{\text{e,h}}^{\text{dir}}+E_{\text{e,h}}^{\text{pol}}$
A(2,1)B(0,0)	A(1,1)B(1,0)	$E_{\text{e,h}}^{\text{dir}}+E_{\text{e,h}}^{\text{pol}}$	$E_{\text{e,h}}^{\text{dir}}+E_{\text{e,h}}^{\text{pol}}$	0
A(2,1)B(0,0)	A(2,0)B(0,1)	$E_{\text{e,h}}^{\text{dir}}+E_{\text{e,h}}^{\text{pol}}$	$-(E_{\text{e,h}}^{\text{dir}}+E_{\text{e,h}}^{\text{pol}})$	$-2(E_{\text{e,h}}^{\text{dir}}+E_{\text{e,h}}^{\text{pol}})$
A(1,1)B(1,1)	A(0,1)B(2,1)	$2(E_{\text{e,h}}^{\text{dir}}+E_{\text{e,h}}^{\text{pol}})$	$E_{\text{e,h}}^{\text{dir}}+E_{\text{e,h}}^{\text{pol}}$	$-(E_{\text{e,h}}^{\text{dir}}+E_{\text{e,h}}^{\text{pol}})$

Supplementary Table S2: The fit parameters obtained from simulations including disorder in the inter-particle distance.

d distribution (nm)	Least squares (a.u.)	τ_{tr} (ps)	μ (cm^2/Vs)
0.3	12.1	69.4	13.7
[0, 0.5]	12.8	69.8	4.88
[0, 1]	12.0	71.0	1.46
[0, 2]	10.1	69.1	0.48

Supplementary Notes

Supplementary Note 1: Coulomb interaction energy

To estimate the dissociation energy of a photogenerated electron-hole pair we first consider the energy E_∞ of a well separated electron-hole pair where the electron and hole occupy different QDs and do not interact. With respect to the unexcited QD film the energy is:

$$E_\infty = E_{1e} - E_{1h} + E_{e}^{pol} + E_{h}^{pol} \quad \text{(S1)}$$

Here E_{1e} and E_{1h} are the site energies of the electron and hole, respectively and E_{e}^{pol} and E_{h}^{pol} are the electron and hole self energies. When the electron and hole are brought together on a single QD the energy becomes:

$$E_\infty = E_{1e} - E_{1h} + E_{e}^{pol} + E_{h}^{pol} + E_{e,h}^{dir} + E_{e,h}^{pol} \quad \text{(S2)}$$

$E_{e,h}^{dir}$ is the direct Coulomb interaction between electron and hole and $E_{e,h}^{pol}$ is the interaction of the electron with the polarization induced by the hole, and *vice versa*. The dissociation energy ΔE_{diss} is obtained as the difference between E_∞ and $E_{e\text{-}h}$ and contains the direct Coulomb interaction $E_{e,h}^{dir}$ between electron and hole, as well as the cross-polarization energy $E_{e,h}^{pol}$.

Expressions for these contributions have been derived by Delerue resulting in the following final expression for the dissociation energy:[5]

$$\Delta E_{diss} = \frac{1.79e^2}{4\pi\varepsilon_0\varepsilon_{in}a} + \frac{e^2}{4\pi\varepsilon_0 a}\frac{\varepsilon_{in}-\varepsilon_{out}}{\varepsilon_{in}\varepsilon_{out}} \quad \text{(S3)}$$

where ε_0 is the vacuum permittivity, a is the QD radius, ε_{in} is the dielectric constant of PbSe QDs, and ε_{out} is the dielectric constant outside of the QDs. ε_{out} is determined by the capping molecules, in this case 1,2-ethanediamine, and neighboring QDs. We consider it to be the effective dielectric constant of the film and obtain an estimate of its value by applying the Bruggeman effective medium theory:[6]

$$f\frac{\varepsilon_{in}-\langle\varepsilon\rangle}{\varepsilon_{in}+\kappa\langle\varepsilon\rangle} = (f-1)\frac{\varepsilon_{m}-\langle\varepsilon\rangle}{\varepsilon_{m}+\kappa\langle\varepsilon\rangle} \quad \text{(S4)}$$

where f is the fill factor of the QDs, $\langle\varepsilon\rangle$ is the effective dielectric function of the film, *i.e.* $\langle\varepsilon\rangle = \varepsilon_{out}$ and ε_m the dielectric function of the capping material. For QDs with a radius of 2.2 nm, a capping layer of 0.2 nm and a total packing density of 0.7, the fill factor is 0.54. With the optical dielectric constant of PbSe (23.9) and EDA (2.11) this results in a ε_{out} of 10.2 and an ΔE_{diss} of 80 meV. This value agrees with the experimental observation of an electron-hole interaction energy of 80 meV in films of PbSe/CdSe core-shell QDs by Swart et al.[7]

Following the same procedure as above, we estimate the Coulomb energy change $\Delta E_{Coulomb}$ for a carrier hopping between any combination of carriers. The site energy, including the first and second terms of Supplementary Equation (S2), and electrostatic self-energy, will not contribute to $\Delta E_{Coulomb}$, so for simplicity we temporarily only consider the direct Coulomb interaction term $E_{e,h}^{dir}$ and the cross-polarization energy $E_{e,h}^{pol}$. The sum of these two terms is referred to as E'.

Supplementary Figure S9**a** shows E' for an exciton. Supplementary Figure S9**b** shows E' for a trion (*eeh*), where we assume that the interactions between two charge carriers are not influenced by the presence of other charge carriers. As a result, in Supplementary Figure S9, the interaction between particle 1 and 2 leads to an energy of $E_{e,h}^{dir} + E_{e,h}^{pol}$, the interaction between 1 and 3 leads to another

$E_{\mathrm{e,h}}^{\mathrm{dir}} + E_{\mathrm{e,h}}^{\mathrm{pol}}$, and the interaction between 2 and 3 gives $E_{\mathrm{h,h}}^{\mathrm{dir}} + E_{\mathrm{h,h}}^{\mathrm{pol}}$. Between two electrons or two holes, the Coulomb repulsion and cross polarization have the same absolute values as for an electron and a hole, but the signs are opposite. To be clear, it is written down here as $E_{\mathrm{e,e}}^{\mathrm{dir}} = E_{\mathrm{h,h}}^{\mathrm{dir}} = -E_{\mathrm{e,h}}^{\mathrm{dir}}$ and $E_{\mathrm{e,e}}^{\mathrm{pol}} = E_{\mathrm{h,h}}^{\mathrm{pol}} = -E_{\mathrm{e,h}}^{\mathrm{pol}}$. By this approach, E' can be calculated for any carrier combination. By comparing E' before and after charge carrier hopping, $\Delta E_{\mathrm{Coulomb}}$ can be calculated, which is presented in Supplementary Table S1.

Supplementary Equation (S3) shows that the dissociation energy and the dielectric constants are directly related. A complexity comes from the fact that the dielectric constant is frequency dependent. In bulk semiconductors, the exciton binding can be determined experimentally, and the proper value of the dielectric constant can be determined.[8] With the magnitude of the exciton binding energy the angular frequency of the electron and hole changes. If this frequency is higher than the frequency of the optical phonons the use of the optical dielectric constant is appropriate, if the frequency is below the optical phonon frequencies, the static dielectric constant applies[8]. As mentioned in the main text, we assume that for QDs the use of the optical dielectric constant is always more appropriate. This results from the fact that electrons and holes always have a high kinetic energy as a result of quantum confinement and the associated frequency will be higher than the optical phonon frequencies[9]. However, using the Monte Carlo simulation we may still evaluate the effect of using various values of the dielectric constants.

With the static ε of 250 for PbSe and 13.8 for 1,2-ethanediamine, the dissociation energy is ~10 meV; with optical frequency values of 23.9 for PbSe and 2.1 for 1,2-ethanediamine, the dissociation energy is ~80 meV.

Supplementary Note 2: Influence of disorder in the inter-particle distance

A uniform distribution is used to describe the variation of the inter-particle barrier width d (*i.e.* dot edge-to-edge distance), and the hopping rate is expressed as: [10, 11]

$$\Gamma_{i\to j} = \Gamma_0 (1 - n_j / g)\exp(-\beta d)\begin{cases} 1 & , \quad E_i > E_j \\ \exp\left(\dfrac{E_i - E_j}{k_{\mathrm{B}}T}\right), & E_i < E_j \end{cases} \tag{S5}$$

where β is the tunneling decay paramter with value of 8.7 ns^{-1} as determined previously.[12]

The values of energy disorder w the exciton dissociation energy and the trion Auger recombination rate are kept constant at 50 meV, 80 meV and ~1/(70 ps), respectively. Supplementary Table S2 and Supplementary Figure S7 show that the coupling disorder decreases the fitted charge carrier mobility and has a small effect on the quality of the simulations.

Supplementary Note 3: Estimate of charge carrier density in solar cells and QD LEDs

In QD solar cells, a short circuit current density with tens mA/cm^2 is frequently measured with a QD solid thickness of several hundred nanometers.[13, 14, 15, 16, 17] A 20 mA/cm^2 current density corresponds to a charge cross-section flow rate of 1.2×10^{17} electrons/(cm^2 s). With a thickness of L =300 nm QD solid, the number of charge carriers flowing through per unit of space per second is 4.0×10^{21} electrons/(cm^3 s). For a QD solid with a fill factor of 55% estimated above and a QD diameter of 4.4 nm, the QD density is 1.2×10^{19} QD/cm^3. Hence, in each second, there are about 330 electrons flowing through every QD. With a charge carrier mobility of μ =1 cm^2/Vs, the residence time for the charge carrier in the solid

is $t_r = L^2/(6\mu^* k_B T) = 5.8\,\text{ns}$. The charge carrier density, which is the product of the residence time and the electron flow rate of 330 electrons/(QD s), is about 2×10^{-6} electrons/QD.

The QD LED in ref.[18] has a reported luminance of 23040 cd/m^2 at 637 nm. The light radiant power is calculated from the luminous intensity as:

$$I_e(\lambda) = \frac{I_v(\lambda)}{683y(\lambda)} \qquad \text{(S6)}$$

where $I_e(\lambda)$ is the radiant intensity (in unit of watts per steradian), I_v is the luminous intensity (in unit of candelas), and $y(\lambda)$ is the standard luminosity function.[19] The radiant power for the above luminous intensity is:

$$I_e(637nm) = \frac{23040*4\pi}{683*0.2} = 168(\text{W/m}^2) \qquad \text{(S7)}$$

corresponding to a photon flux is $5.4\times10^{20}(\text{m}^2\text{s})^{-1}$. With a radiative emission rate of 1/(30 ns), and 2 monolayers of quantum dots with 4 nm diameter,[18] the exciton density is estimated as:

$$n = \frac{5.4\times10^{20}\times30\times10^{-9}}{2/(16\times10^{-18})} = 1.3\times10^{-4}(\text{excitons/QD}) \qquad \text{(S8)}$$

Supplementary Methods: Monte Carlo simulation details

Monte Carlo simulations were performed on a computer cluster using a program written in Matlab. The flow chart of the simulations is:

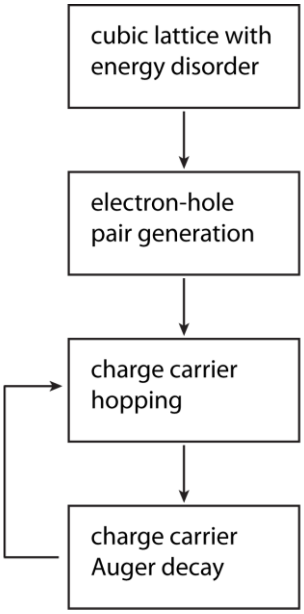

We assume that the QDs are situated on a regular cubic lattice. The lattice size is $50\times50\times50$ for most of the simulations except for the charge carrier mobility simulation where a size of $200\times200\times200$ is used. The energy levels of electrons and holes are subject to disorder and are drawn from a Gaussian density of states,

$$g(E) = a\exp\left(-\frac{(E-E_0)^2}{(2w)^2}\right) \tag{S9}$$

where a is the normalizing constant, and E_0 is an arbitrary energy since it does not contribute to the hopping rate. Positional disorder is not included for most of the simulations, although its effect is discussed below. Electrons and holes behave identically in these simulations: their density of states and the attempt hopping rates are the same.

Photoexcitation of an electron-hole pair was modeled by assigning a number pair (1,1) on a site in the lattice, and two electron-hole pairs (2,2) are generated by assigning (1,1) two times on the same site, and so on. For an excitation density of $<N_{\mathrm{abs}}>$, the number pair (1,1) was randomly assigned to a site, which was repeated for $<N_{\mathrm{abs}}>\times(\text{number of sites})$ times. As a consequence, the distribution of the electron-hole pairs on the lattice follows a Poisson distribution $P_{(\mathrm{n,n})} = <N_{\mathrm{abs}}>^n \exp\left(-<N_{\mathrm{abs}}>\right)/n!$.

After the initial charge carrier generation, the carrier hopping process is modeled by repopulating the charge carriers one by one according to their hopping probabilities. For a charge carrier situated on a site i, in a time interval it may hop to one of its six nearest neighbors sites j. This was modeled by generating a random "decision" number according to its staying and hopping probabilities (*i.e.* seven decision numbers for a cubic lattice). The probability for a specific hopping event from site i to j is:

$$P_{\mathrm{i}\to\mathrm{j}} = \left(1-\exp(-\delta t\sum_{ii=1,\ldots,6}\Gamma_{i\to ii})\right)\frac{\Gamma_{i\to j}}{\sum_{ii=1,\ldots,6}\Gamma_{i\to ii}} \tag{S10}$$

$$\Gamma_{i\to j}=\Gamma_0(1-n_j/g)\begin{cases}1 & , \quad E_i>E_j\\ \exp\left(\dfrac{E_i-E_j}{k_\mathrm{B}T}\right), & E_i<E_j\end{cases} \tag{S11}$$

The sum runs over the six nearest neighbor sites $ii=1,\ldots,6$ of the site i, and δt is the simulation time interval with a small value that is less than 10 fs. The term within the bracket in Supplementary Equation (S10) describes the total hopping probability of a charge carrier in the time interval δt, while the second fractional term is the chance of hopping to the site j if a hopping process occurs. The energy change (E_i-E_j) in Supplementary Equation (S11) includes contributions of site energy and the Coulomb interaction energy (see Supplementary note 1). The charge carrier decay via Auger recombination was modeled by removing electron-hole pairs site by site according the Auger decay probabilities

$$P_i^{\mathrm{AR}}=1-\exp\left(-\delta t k_{i,(n_\mathrm{e},n_\mathrm{h})}\right) \tag{S12}$$

where $k_{i,(n_\mathrm{e},n_\mathrm{h})}$ is the Auger decay rate given by Supplementary Equation (2) in the main text. After the removing of the charge carriers, the information of the charge carrier population was extracted, and the simulation proceeded to the next step of the hopping process simulations, as shown in Supplementary Figure S8.

The charge carrier mobility μ is related to the diffusion coefficient D via the Einstein-Smoluchowski relation:

$$\mu=\frac{e}{k_\mathrm{B}T}D \tag{S13}$$

and the diffusion coefficient can be determined by simulating the mean squared displacement $\langle r^2\rangle$ of charge carriers as a function of time t:[20]

$$\langle r^2\rangle=2nDt \tag{S14}$$

where n is the dimensionality of the system (*i.e.* n=3 here).

The mean squared displacement is determined by simulations of a single charge carrier diffusing in the system. Supplementary Figure S10 shows $\langle r^2\rangle$ as a function of time for various values of the energy disorder w. The linear region is used to determine the diffusion coefficient via Supplementary Equation (13). The fact that for high values of w the initial slope is higher results from thermalization of the charge carrier in the disordered DOS and a concomitant reduction in mobility[21].

Supplementary References

38 Hynninen, A. P., Filion, L. & Dijkstra, M. Stability of LS and LS2 crystal structures in binary mixtures of hard and charged spheres. *J. Chem. Phys.* **131**, 064902 (2009).

39 Evers, W. H. *et al.* Entropy-Driven Formation of Binary Semiconductor-Nanocrystal Superlattices. *Nano Lett.* **10**, 4235-4241 (2010).

40 Delerue, C. & Lannoo, M. *Nanostructures theory and modeling*. (Springer, 2004).

41 Bruggeman, D. A. G. *Ann. Phys.* **24**, 636–664 (1935).

42 Swart, I., Sun, Z. X., Vanmaekelbergh, D. & Liljeroth, P. Hole-Induced Electron Transport through Core-Shell Quantum Dots: A Direct Measurement of the Electron-Hole Interaction. *Nano Lett.* **10**, 1931-1935 (2010).

43 Knox. *Theory of excitons*. (Academic press, 1963).

44 Lippens, P. E. & Lannoo, M. Comparison between Calculated and Experimental Values of the Lowest Excited Electronic State of Small Cdse Crystallites. *Phys. Rev. B* **41**, 6079-6081 (1990).

45 Leschkies, K. S., Beatty, T. J., Kang, M. S., Norris, D. J. & Aydil, E. S. Solar Cells Based on Junctions between Colloidal PbSe Nanocrystals and Thin ZnO Films. *ACS Nano* **3**, 3638-3648 (2009).

46 Choi, J. J. *et al.* PbSe Nanocrystal Excitonic Solar Cells. *Nano Lett.* **9**, 3749-3755 (2009).

47 Luther, J. M. *et al.* Schottky Solar Cells Based on Colloidal Nanocrystal Films. *Nano Lett.* **8**, 3488-3492 (2008).

48 Ma, W., Luther, J. M., Zheng, H. M., Wu, Y. & Alivisatos, A. P. Photovoltaic Devices Employing Ternary PbSxSe1-x Nanocrystals. *Nano Lett.* **9**, 1699-1703 (2009).

49 Pattantyus-Abraham, A. G. *et al.* Depleted-Heterojunction Colloidal Quantum Dot Solar Cells. *ACS Nano* **4**, 3374-3380 (2010).

50 Kwak, J. *et al.* Bright and Efficient Full-Color Colloidal Quantum Dot Light-Emitting Diodes Using an Inverted Device Structure. *Nano Lett.* **12**, 2362-2366 (2012).

51 Sharpe, L. T., Stockman, A., Jagla, W. & Jagle, H. A luminous efficiency function, V*(lambda), for daylight adaptation. *J. Vision* **5**, 948-968 (2005).

52 Roque-Malherbe, R. M. *Adsorption and diffusion in nanosparous material.*, (CRC press., 2007).
